# Untargeted metabolic analysis in serum samples reveals metabolic signature in children with congenital heart failure on enalapril therapy

**DOI:** 10.3389/fped.2025.1530063

**Published:** 2025-04-28

**Authors:** N. J. L. Smeets, I. N. van Hoek, J. J. M. Jans, M. Dalinghaus, S. Laer, M. Bajcetic, C. Male, S. N. de Wildt

**Affiliations:** ^1^ Department of Pharmacology and Toxicology, Radboud Institute of Health Sciences, Radboud University Medical Center, Nijmegen, Netherlands; ^2^ Department of Genetics, Section Metabolic Diagnostics, University Medical Center Utrecht, Utrecht, Netherlands; ^3^ Department of Pediatric Cardiology, Erasmus MC – Sophia, Rotterdam, Netherlands; ^4^ Institute of Clinical Pharmacy and Pharmacotherapy, Heinrich-Heine-University Düsseldorf, Düsseldorf, Germany; ^5^ Department of Clinical Pharmacology, University Children’s Hospital, Belgrade, Serbia; ^6^ Department of Pharmacology, Clinical Pharmacology and Toxicology, School of Medicine, University of Belgrade, Belgrade, Serbia; ^7^ Department of Paediatrics and Adolescent Medicine, Medical University of Vienna, Vienna, Austria; ^8^ Intensive Care and Department of Pediatric Surgery, Erasmus MC Sophia Children’s Hospital, Rotterdam, Netherlands

**Keywords:** heart failure, enalapril, pharmacokinetics, children, ACE inhibitor

## Abstract

**Introduction:**

Enalapril is an angiotensin-converting enzyme (ACE) inhibitor (ACEi) which is widely used in the management of (paediatric) hypertension and heart failure (HF). There is a significant interindividual variability in the patient's response to enalapril that is not completely understood. Therefore, we aimed to examine the potential of metabolic profiling for stratifying paediatric patients with HF due to congenital heart disease (CHD) in terms of treatment response to enalapril. Additionally, we investigated metabolic profiles in CHD patients and healthy controls.

**Methods:**

CHD patients aged 0–6 years of age who previously participated in a multi-centre and multinational pharmacokinetic safety bridging study of enalapril were included. Patients were defined as responder when aldosterone levels decreased after a single administration of enalapril. Non-responders were those with an increase in their aldosterone levels. We applied an untargeted mass spectrometry-based metabolomics approach on serum. By using both supervised and unsupervised learning algorithms, we compared metabolic profiles between responders and non-responders as well as between patients and age and sex matched healthy controls.

**Results:**

In total, 63 patients were included with a median age of 132 (IQR 54–211) days and 46 controls [97 (63–160) days]. 41 of 63 patients responded to enalapril therapy. Their baseline characteristics were similar to non-responders (*n* = 22). A total of 1,820 unique features were identified. Responders were distinguished from non-responders using a supervised learning algorithm based on 94 features (*p* = 0.05). Furthermore, metabolic profiles could distinguish between patients and controls based on an unsupervised learning algorithm which revealed 278 relevant features (*p* = 0.001).

**Conclusions:**

These are the first data to demonstrate a clear metabolic signature in children with CHD using ACEi. We identified metabolites whose concentrations were both associated with ACEi response and HF. This indicates more severe HF in patients with more profound treatment response. Our results will therefore allow further studies aiming at disentangling variability in ACEi treatment response.

## Introduction

Enalapril is an angiotensin-converting enzyme (ACE) inhibitor (ACEi) which is widely used in the management of (paediatric) hypertension and heart failure (HF). It exhibits its effect after hepatic metabolism to its active metabolite: enalaprilat. Enalaprilat inhibits ACE, leading to a decrease in formation of angiotensin II (ATII) and a diminished aldosterone secretion. This results in downregulation of the Renin Angiotensin Aldosterone System (RAAS), thereby decreasing blood pressure (BP) and reducing the cardiac afterload (1), ultimately improving left ventricle function (2). In adults, there is significant interindividual variability in the patient's response to ACEi based on both physiological (e.g., BP) and biochemical (e.g., RAAS markers) parameters, and 50% of hypertensive patients still fail to achieve target BPs (3). This variability in treatment response is not completely understood, even when taking factors potentially influencing drug disposition like disease (4–7), food intake (8) and race (9) into account. As the moderate effectiveness of ACEi therapy and unexplained variability in therapy remained over the past decade, innovative approaches were pursued in order to better understand treatment response and ultimately improve effectiveness.

Metabolomics is increasingly used to understand variation to treatment response (10). This is the analysis of small molecules within a biologic specimen of which the concentration is a reflection of all the biochemical processes in one individual. Because it accounts for many sources of variation, including those in the genome, transcriptome and proteome, it aids in understanding disease and response to therapy (3, 11). In the adult population, genotype dependent differences in the metabolomic response to ACEi intake were investigated and several dipeptides were considered as potential functional markers of ACE activity in adult patients, regardless of indication (11). Also, metabolites could predict a poor response to lisinopril—which is also an ACEi—in the treatment of hypertension (10) or were related to ACE-activity (11). Thus, discovered metabolites offer leads in predicting treatment response and individualize treatment in adults.

Paediatric HF, however, differs from adult HF with regards to aetiology, clinical manifestation, comorbidities and prevalence (12). Also, there is an increased activity of the RAAS during infancy and childhood (up to four years of age) (13). Therefore, adult findings cannot simply be extrapolated to the paediatric population. To the best of our knowledge, no metabolomic studies have been conducted in paediatric patients treated with ACEi.

In a multicentre, multinational Phase II/III prospective, open-label pharmacokinetic bridging study (LENA), the pharmacokinetics (PK), pharmacodynamics (PD) and safety of enalapril were investigated in children with HF due to congenital heart disease (CHD) (14). Results showed interindividual variability in response to treatment (15). As adult metabolomic studies have helped in disentangling treatment response to ACEi, we aimed to examine the potential of metabolic profiling for stratifying paediatric patients with CHD in terms of treatment response, ultimately aiming at individualizing treatment. We additionally investigated metabolic profiles in healthy paediatric controls and compared those to profiles patients with CHD-related HF to better understand this condition. A small number of paediatric studies investigated metabolic profiles in variety of diseases and comparing patient profiles to healthy controls has led to a better understanding of a wide variety of diseases. This could offer the opportunity to identify diagnostic or therapeutic biomarkers.

## Methods

### Patient characteristics

This exploratory metabolomic study was part of a multicentre, multinational Phase II/III prospective, open-label, pharmacokinetic bridging study in patients with CHD (LENA) (14). In short, study patients were treated with newly developed orodispersible minitablets (ODMT) of enalapril and systematically assessed for PK and pharmacodynamics (PD), as well as clinical parameters. For this metabolomic study, only CHD patients aged 0–6 years of age whose parents specifically consented for this sub study could be included.

Among inclusion criteria were a diagnosis of HF due to CHD, need for afterload reduction by drug therapy and a bodyweight greater than 2.5 kg. Patients needed to be naive to ACEi or already on ACEi but willing to switch to enalapril ODMTs. Patients were excluded when using dual ACEi therapy, renin inhibitors, ATII antagonists and non-steroidal anti-inflammatory drugs. Patients with a BP below the fifth percentile for age were also excluded. The full list of in- and exclusion criteria was listed in the study protocol (14). A minimum of 60 patients needed to be included with at least 37 patients below the age of 12 months in order to obtain adequate paediatric PK data and to accurately describe the dose-exposure in this population. For this metabolomic study, control samples were derived from sex and age matched healthy children, of which blood samples were obtained before any small medical procedure or surgery.

### Ethical approval

For the initial LENA study (including metabolomics substudy), the medical ethics review board of the responsible centers approved the protocol. This included the Univerzitetska Dečja Klinika in Belgrade, Serbia, the Medizinische Universität in Wien, Austria, the Göttsegen Gyorgy Orszagos Kardiologiai Intezet HPHC in Budapest, Hungary, the Erasmus Medical Center in Rotterdam, the Netherlands, the Wilhelmina Kinderziekenhuis of the University Medical Centre in Utrecht, The Netherlands as well as the Great Ormond Street Hospital for Children, NHS Trust GOSH in London, United Kingdom. For the collection of healthy control samples, the Erasmus Medical Center medical ethics review board approved the protocol. Written informed consent was obtained from all subjects' families before enrolment.

### Study procedures

Enalapril was administered orally as ODMTs and intake at the initial dose visit took place at the clinic. Patients naive to ACEi therapy were uptitrated according to a defined dose titration scheme (Supplementary Table S1) and patients who were already on enalapril remained on the same dose when switched to the enalapril ODMTs. Clinical parameters assessed at the initial dose visit included the Ross score for accurate grading of the presence and severity of paediatric HF (range: 0–12) (16), shortening fraction (SF) based on echocardiography and non-invasive mean arterial pressure (BP). Blood was withdrawn for RAAS activity [including renin, angiotensin I (ATI), aldosterone & plasma renin activity (PRA)] before administration and 4 h after administration of enalapril. Also, a full PK curve was collected with sampling points at 1, 2, 4, 6 & 12 h after administration. For metabolomic analysis, blood was withdrawn before enalapril administration.

### Laboratory analysis

PD and PK markers, including renin, PRA, ATI, enalapril and enalaprilat concentrations were measured as previously described (17). In short, enalapril and enalaprilat concentrations were determined using high-performance liquid chromatography. The enalapril and enalaprilat pharmacokinetics were characterized by the maximum serum concentration (C_max_) as well as area under the curve (AUC) from zero to infinity. This AUC was calculated by the trapezoidal rule with infinity extrapolation (17). Renin was determined using a chemiluminescent immunoassay based on monoclonal antibodies. PRA and ATI were determined in a ^125^I radioimmunoassay using ATI antibodies and circulating immunoreactive ATI. Aldosterone was measured by a previously validated immunoassay. This assay was validated in the aldosterone calibration range between 43 and 958 pg/ml. The mean difference between the original and repeat of the incurred LENA sample reanalysis was –4% (18).

### Metabolic sample preparation and profiling

Metabolites were analysed in serum by, a semi-quantitative direct-infusion high-resolution mass spectrometry-based metabolomics method in combination with a nano-electrospray ionization source, as described previously (19). Briefly, metabolites were extracted from 7.5 µl of serum by adding 140 µl methanol with stable isotope labeled internal standards (acylcarnitines and amino acids). This solution was centrifuged for five minutes at 17,000 g and the supernatant diluted with 45 µl 0.3% formic acid. This was filtered using a methanol preconditioned 96 well filter plate (Acro prep, 0.2 m GHP, NTRL, 1 ml well; Pall Corporation, Ann Arbor, MI, USA) and a vacuum manifold. The sample filtrate was collected in a 96 well plate (Advion, Ithaca, NY, USA). Samples (13 µl) were injected in triplicate into the Q-Exactive high-resolution mass spectrometer using a TriVersa NanoMate system (Advion, Ithaca, NY, USA) controlled by Chipsoft software (version 8.3.3, Advion). The Q-Exactive high-resolution mass spectrometer was operated in positive and negative ion mode in a single run, with automatic polarity switching. There were two time segments of 1.5 min with a total run time of 3.0 min. Scan range was 70–600 mass to charge ratio (m/z), resolution was 140,000 at m/z = 200. Data acquisition was performed using Xcalibur software (Thermo Scientific™, Waltham, MA, USA). Using MSConvert15 (ProteoWizard Software Foundation, Palo Alto, CA, USA), raw data files containing scanning time, m/z and peak intensity were converted to mzXML format. Data processing and peak calling was done using an in-house developed pipeline (https://github.com/UMCUGenetics/DIMS). Detected mass peaks were annotated by matching the m/z of the mass peak with a range of five parts per million to metabolite masses present in the Human Metabolome Database (HMDB) (19). Metabolite annotations without adduct ions in negative or positive mode ([M - H]^–^,[M + H]^+^), or with the single adduct ions [M + Na]^+^, [M + K]^+^, and [M + Cl]^−^ were selected For each sample, the intensities of these five mass peaks were summed, resulting in one (summed) mass peak intensity per metabolite annotation: ∼6,600 summed mass peaks in total. Next, exogenous and drug metabolite annotations were excluded resulting in ∼3,900 summed mass peaks in total, corresponding to ∼1,900 metabolite (isobaric compounds) annotations, since mass peaks can account for several isomers. For each mass peak per patient sample, the deviation from the intensities in control samples was indicated by a *Z*-score, calculated by: *Z*-score=(intensity patient sample—mean intensity control samples)/standard deviation intensity control samples.

### Data analysis

Patients were categorized as responder or non-responder based on the difference between their pre and post-dose aldosterone levels. Understanding the difference in aldosterone response is of value as this is the final and direct effector of the RAAS, and, independent of BP, its levels are associated with clinical outcomes in children (20). As inhibition of the RAAS by enalapril would normally lead to decreased aldosterone levels, patients were defined as responders when their aldosterone levels decreased after administration of enalapril. The difference in BP after enalapril administration was not used to categorise patients as accurate BP measurements in infants is prone to error and therefore less reliable. Differences between groups were assessed using a Mann–Whitney *U*-test. For continuous variables, data were expressed as median values with interquartile ranges (IQR) when not normally distributed. For comparison between PK and PD markers prior to and after administration, a Wilcoxon signed rank test was used. To display categoric variables, numbers and percentages were used. Statistical analysis took place using SPSS version 25.0.

MetaboAnalyst was used to analyse metabolic features. The majority of metabolites are isobaric compounds and do not have unique nominal masses. With direct infusion mass spectrometry, these isobaric compounds cannot be separated and a feature can therefore represent one or multiple metabolites. Both principal component analysis (PCA) (unsupervised learning algorithm) and partial least-squares discriminant analysis (PLS-DA) (supervised learning algorithm) were employed to visualize and interpret differences, aiming to distinguish responders from non-responders and patients from healthy controls. For feature selection, e.g., selection of features that differed significantly between two groups, a two sample *T*-test on raw data was applied. Data were not filtered as the number of features did not exceed 5,000 and all features were included in the analysis. We did not correct for multiple testing To achieve clearer results, data ware scaled using auto scaling (mean-centred and divided by the standard deviation of each variable). Assuming equal group variance as many biological measurements have a typical pattern of dispersion with a normal distribution, two-sample *T*-tests were used to determine metabolites of interest to differentiate between groups. For all identified important features, the relation to cardiovascular diseases for the identified metabolite, but also for its isobaric compounds were investigated (21). Last, to potentially link unknown metabolic features, a clustering analysis was conducted to reveal up- or downregulated pathways between groups.

### Data sharing statement

Our raw data were published at MetaboLights, which is a database for Metabolomics experiments and derived information. *(Link will follow after acceptation)*.

## Results

### Patient characteristics

In total, 67 patients were included in the LENA study and all gave consent for participation in this metabolomic study. Of these 67 patients, almost half (*n* = 31) were diagnosed with an AVSD (46%), 21 with a VSD (31%), three with mitral valve insufficiency (5%), eleven patients had a variety of diagnosis including a complete AV-canal defect, a double outlet right ventricle, hypoplastic left heart syndrome, patent arterial duct and transposition of the great arteries. Of one patient, the diagnosis was not registered and could not be recalled. Serum samples were collected, and for 63 of these patients, aldosterone (before and after administration of enalapril) and metabolite levels (before administration only) were available. Based on differences between aldosterone levels prior to and after enalapril administration, responders (*n* = 41) and non-responders (*n* = 22) were defined. Median change in aldosterone levels was−144.3 (IQR −353.9 to −60.1 pg/ml) for responders, whereas non-responders had a median change of 61.2 (28.4–210.4) pg/ml. Both patient groups had similar baseline characteristics prior to enalapril therapy (Table 1). Ross scores of both groups indicate mild HF, while SFs are within normal paediatric ranges (between 29% and 46%, lower percentages indicating worse LV function). There was an equal decrease in BP after enalapril administration, four hours post dose, in responders (median −3.3, IQR −9.5 to 1.3 mmHg) and non-responders (median, −5.3, IQR −12.1 to 6.8 mmHg) (Wilcoxon signed rank, *p* = 0.005 and *p* = 0.042, respectively). Although statistically not significant, median initial aldosterone levels were higher in responders [576 (IQR 249–965) pg/ml] compared to non-responders [434 (IQR 94–731)] (*p* = 0.055).

**Table 1:** Baseline characteristics of patients (responders and non-responders) and healthy controls.

	Responders (*n* = 41)	Non-responders (*n* = 22)	*P*-value	Healthy controls (*n* = 46)
Demographics pre dose				
Age (days)^a^	117 (49–195)	133 (55–251)	0.498	97 (63–160)
Weight (kg)^a^	4.7 (3.8–6.5)	5.0 (3.9–7.0)	0.773	–
Percentage male: no (%)	20 (49%)	12 (55%)	–	28 (61%)
Pretreated with ACEi: no (%)	19 (46%)	13 (59%)	–	–
Concomitant use of spironolactone	34 (83%)	18 (82%)	–	–
Shortening fraction^a^	0.39 (0.35–0.45)	0.41 (0.37–0.45)	0.435	–
Ross score^a^	3.0 (2.0–6.0)	4.5 (1.8–7.0)	0.586	–
BP (mmHg)^a^	69 (61–75)	66 (61–75)	0.579	–
Pharmacokinetic measurements				
Enalapril starting dose (mg/kg)^a^	0.12 (0.09–0.15)	0.14 (0.10–0.15)	0.249	–
Enalapril C_max_ (ng/ml/mg × kg)^a^	260 (159–430)	262 (136–326)	0.558	–
Enalapril AUC (ng/ml × h/mg × kg)^a^	777 (494–1,033)	769 (407–1,232)	0.887	–
Enalaprilat C_max_ (ng/ml/mg × kg)^a^	116 (76–180)	142 (84–204)	0.414	–
Enalaprilat AUC (ng/ml × h/mg × kg)^a^	961 (556–1,431)	1,147 (676–1,717)	0.378	–
Pharmacodynamic measurements prior to dose				
Initial renin levels (pg/ml)^a^	197 (75–398)	96 (47–469)	0.270	–
Initial angiotensin I levels (ng/ml)^a^	2.0 (1.0–2.7)	1.5 (0.8–2.3)	0.272	–
Initial aldosterone levels (pg/ml)^a^	576 (249–965)	434 (94–731)	0.055	–
Initial plasma renin activity (ng/ml/h)^a^	34 (11–68)	14 (8–48)	0.141	–
Difference in mean arterial pressure (mmHg), between pre and post dose^a^	−3.3 (−9.5–1.3)	−5.3(−12.1- 6.8)	0.686	–

*P*-value based on Mann–Whitney *U*-test comparing responders vs. non-responders.

^a^ median (IQR).

Control samples were collected from 46 children with a median age of 97 (IQR 63–160) days, this was equal to the median age of our patients [132 (IQR 54–211) days] (Mann–Whitney U, *p* = 0.306).

### Metabolomic analysis

#### Responders vs. non-responders

**Figure 1:**
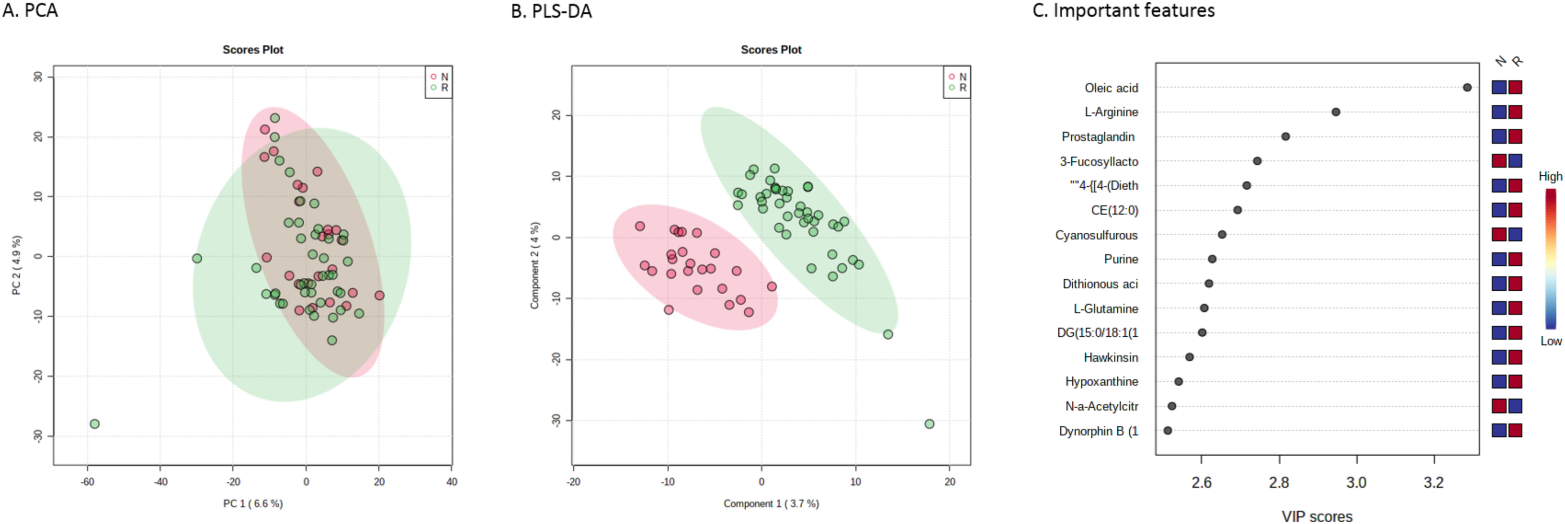
Metabolic profiles of responders and non-responders to enalapril therapy. **(A)** Principal component analysis score plot and **(B)** partial least-squares discriminant analysis serving as visualization for distinguishing responders (in green) and not-responders (in red) to enalapril therapy. **(C)** Important features plot, indicating the 15 most important metabolites that were relevant for distinguishing responders from non-responders. PC, principal component; VIP, variable importance in projection.

First, the metabolic profiles were analysed in patients only, and a total of 1,820 unique features were identified. Unsupervised PCA did not display two distinct clusters of individuals. Using PLS-DA, responders and non-responders differed significantly (Figure 1). A two-sample *T*-test, however, revealed 94 significantly different features (*p*-value threshold of 0.05) between responders and non-responders of which the 15 most important ones (sorted by highest *t*-value) are visualised in Figure 1C (full list of identified features with corresponding isobaric compounds listed in Supplementary Table S1, sorted by *t*-value).

#### Healthy controls vs. CHD patients

**Figure 2:**
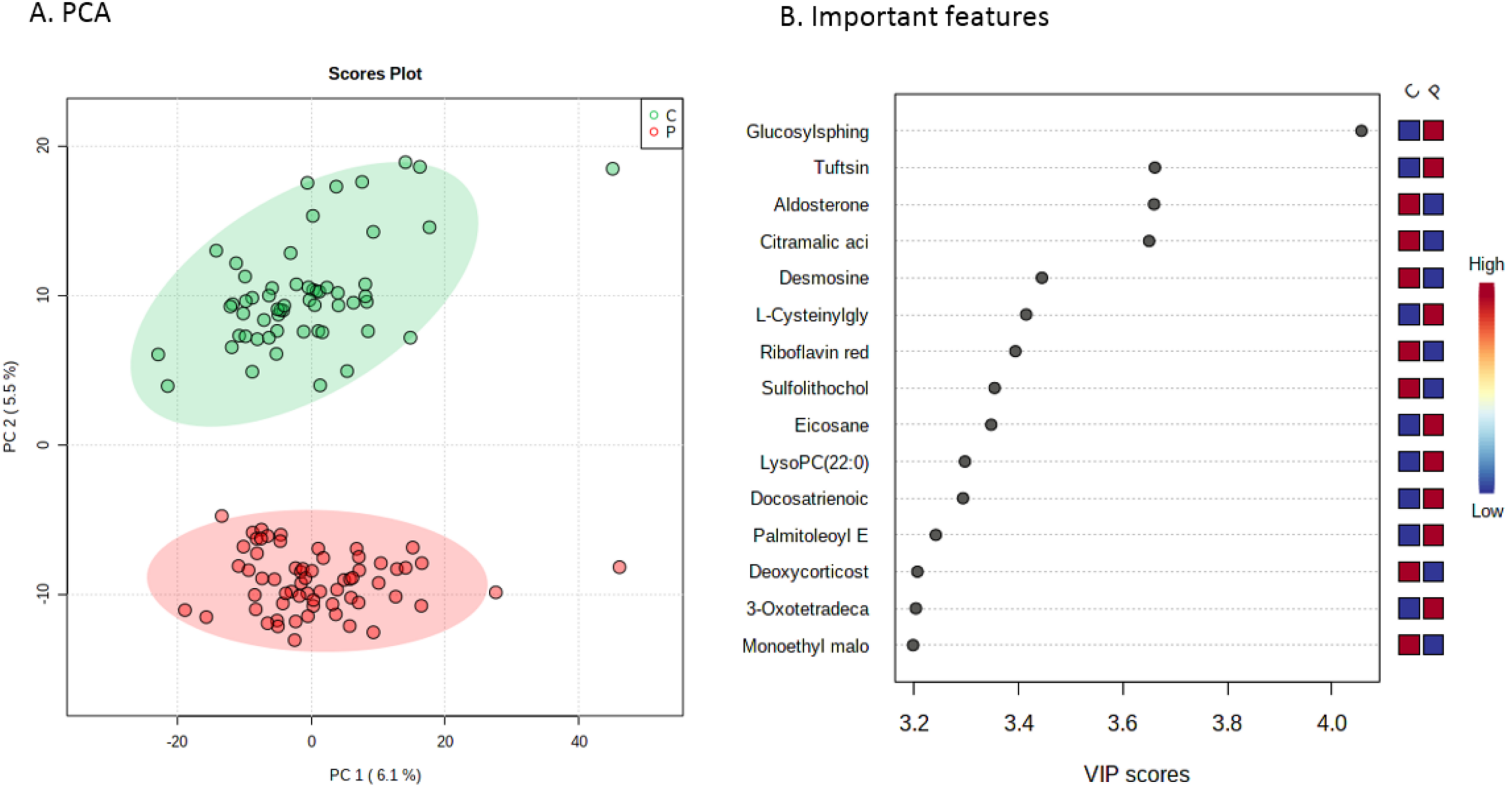
Metabolic profiles of CHD patients vs. controls. **(A)** Principal component analysis score plot serving as visualization for distinguishing patients (in red) from healthy controls (in green). **(B)** Important features plot, indicating the 15 most important metabolites that were relevant for distinguishing patients from controls. PC, principal component; VIP, variable importance in projection.

Next, metabolic profiles in patients were compared to healthy controls. Here, PCA revealed two distinct clusters of individuals (Figure 2). Using again a two-sample *T*-test, this revealed 278 unique features (*p*-value threshold of 0.001), listing the most distinctive features in Figure 2 (full list of features with corresponding isobaric compounds in Supplementary Table S2).

Last, clustering analysis did not reveal (groups of) pathways that were up- or downregulated and could explain differences between groups.

## Discussion

This metabolomic study in paediatric patients with CHD-related HF was designed to identify metabolites that distinguish patients who respond to enalapril therapy based on their aldosterone levels from patients who do not respond. Our untargeted metabolomic approach identified a distinct metabolic signature for patients responding to enalapril therapy. As a secondary objective, the difference in metabolic profiles patients and healthy age-matched controls was assessed to better understand the pathophysiology of CHD-related HF. Our analysis demonstrated a clear difference between patients and healthy controls based on their metabolic profiles.

Responders vs. non-responders some of the metabolites associated with response to enalapril therapy have been previously associated with more severe cardiovascular diseases in other cohorts (Table 2). This includes oleic acid, arginine, prostaglandin F2a and threonine, which were all higher in responders vs. non-responders. Oleic acid has been associated with more severe HF (22) as well as with increased ATII levels in obese hypertensive adults (23). Also, an interaction between oleic acid and ATII led to lower cell viability and an increased number of vascular small muscle cells (24). As higher oleic and ATII levels are both present in patients with a higher cardiovascular risk score, Greene et al. hypothesized that oleic acid and ATII might interact to accelerate vascular disease in obese hypertensive patients. Furthermore, arginine and prostaglandin 2a have been associated with (more severe) HF. There are higher serum concentrations of arginine and reduced myocardial nitrogen oxide production (25), as well as increased prostaglandin F2a and threonine levels in patients with HF (26, 27). Also, although not statistically significant, PD markers of the RAAS were higher in our responders compared to non-responders (*p* = 0.055–0.272) before enalapril administration, suggesting increased RAAS activation in these patients. Because RAAS is activated in high-risk cardiovascular patients, together with the above mentioned associations for metabolites, this might indicate that patients responding to enalapril therapy may have been more severely ill. The definition of responders based on a single aberrant level of aldosterone following a dose of ACE inhibition could constitute a limitation of our study design. After a significant period of heart failure therapy, alternate pathways of aldosterone formation might occur, this is known as the aldosterone escape theory (REF). Yet, we feel that in our population in which the majority of patient were not on heart failure treatment for a long period of time, aldosterone is still the main identifier for treatment response.

**Table 2:** Overview of metabolites that were previously linked to heart failure (severity).

Direction of effect	Metabolite (number in our list)	Relation to heart failure	Reference
Responders vs. non-responders			
R > NR	Oleic acid (1)	Associated with more severe HF (Zhou et al). Associated with increased ATII levels and a higher cardiovascular risk score in obese hypertensive adults (Greene et al)	Zhou et al. (22) Greene et al. (23)
R > NR	Arginine (2)	Higher in HF patients compared to controls	Zordoky et al. (32)
R > NR	Prostaglandin F2a (3)	Higher in HF patients compared to healthy controls	Kotlyar et al. (27) Castellani et al. (26)
R > NS	Glutamine (11)	Lower in HF patients compared to healthy controls	Wang et al. (31)
R > NS	Histidine (33)	Lower in HF patients compared to healthy controls (Wang et al) Higher in HF patients compared to healthy controls (Zordoky)	Wang et al. (31) Zordoky et al. (32)
R > NS	Threonine (35)	Higher in HF patients compared to controls	Zordoky et al. (32)
R > NR	Citrulline (73)	Different between patients with HF stadium B, C and D compared to patients with HF stadium A (unknown which direction)	Zhou et al. (22)
Patients vs. controls			
P > C	Isoleucine leucine (224)	Different between patients with HF stadium B, C and D compared to patients with HF stadium A (unknown which direction) Higher in HF patients compared to controls	Zhou et al. (22) Wang et al. (31)
P > C	Glutamine (50)	Lower in HF patients compared to controls	Wang et al. (31)
P > C	Arginine (68)	Higher in HF patients compared to controls	Zordoky et al. (32)
P > C	Betaine (72)	Higher in HF patients compared to controls	Zordoky et al. (32)
P > C	Dodecanedioylcarnitine (93)	Lower in paediatric HF patients compared to controls	O’Connell et al. (37)
P > C	Proline (126)	Higher in HF patients compared to controls Different between patients with HF stadium B, C and D compared to patients with HF stadium A (unknown which direction)	Wang et al. (31) Zhou et al. (22)
P > C	Glycocholic acid (146)	Different between patients with HF stadium B, C and D compared to patients with HF stadium A (unknown which direction) Higher in paediatric HF patients compared to controls	Zhou et al. (22) O’Connell et al. (37)
P < C	Taurocholic acid (155)	Higher in paediatric HF patients compared to controls	O’Çonnell et al, (37)
P > C	Creatine (168)	Higher in HF patients compared to healthy controls Higher disease severity scores in children undergoing surgery for CHD	Wang et al. (31) Correia et al. (36)
P > C	Carnitine (175)	Higher in HF patients compared to controls	Zordoky et al. (32)
P < C	2-methylglutaric acid (209)	Different between patients with HF stadium B, C and D compared to patients with HF stadium A (unknown which direction)	Zhou et al. (22)

R, responders; NR, non-responders; P, CHD patients; C, healthy controls; CHD, congenital heart disease.

Even though Ross scores and SFs were equal between responders and non-responders, these classifications have limitations. The use of the Ross score remains still need to be validated as a surrogate clinical endpoint in large number of patients, impairing its current value for scoring HF severity in children (28). Also, SF determination relies on normal left ventricle shape, which may be altered in the presence of congenital heart defects (29). Thus, less pronounced differences in disease severity may very well exist between groups, even though Ross scores and SFs indicated mild heart failure only. However, not all metabolites support this finding. Glutamine, which was higher in responders, promoted cardiovascular health by exerting antioxidant and anti-inflammatory effects as well as by optimizing nitric oxide synthesis in adults (30). In accordance, in adult HF patients, glutamine levels were lower (31). For histidine and citrulline, available data are conflicting, suggesting both a negative and positive relationship with HF (22, 31, 32). None of the other features distinguishing responders from -non-responders was associated with cardiovascular disease or RAAS.

### Patients vs. controls

Interestingly, although unsupervised learning clearly separated the CHD patients and controls, none of the top 15 identified metabolites had a known association with cardiovascular disease except for aldosterone, which was lower in patients. Because 50% of all patients were pretreated with ACEi before the start of enalapril therapy, this can explain the lower aldosterone levels in patients vs. controls. All other important features (top 15) were not previously linked to disease, except for glucosylsphingosine, which was significantly lower in patients. This is a well-known marker for Gaucher disease, a rare genetic disorder in which sphingolipids accumulate in cells, leading to immune dysregulation and skeletal disease (33). Yet, why glucosylsphingosine levels are higher in our paediatric patients, needs investigation.

Although the top 15 features do not have a known association with cardiovascular disease, other features further down the list were previously associated with (more severe) HF. Multiple metabolomic studies in adult HF patients (22, 31, 32) revealed a great number of dysregulated metabolites that could be potential biomarkers. Many of the reported metabolites that were associated with (more severe) HF, were also higher in our patients than controls (isoleucine, arginine, betaine, proline, creatine & carnitine (Table 2). For instance, creatine will be elevated in case of muscle damage and was higher in patients vs. controls. Whether this can be attributed to cardiac muscle damage is unknown. Again, for glutamine, which was higher in our patients, levels were lower in HF patients compared to healthy controls (31). Although glutamine levels are significantly lower in children compared to adults (34), this cannot explain the observed differences.

To the best of our knowledge, we are the first to report metabolomic profiles in paediatric patients on ACEi therapy (35).. Three metabolomic studies were conducted in children with HF. Correia et al. investigated metabolic profiles of 28 children undergoing surgery for CHD (36). These had a median Risk Adjustment for Congenital Heart Surgery (RACHS) score of 2 (IQR2-3), with 6 being the highest and 1 the lowest score possible. Patients were slightly older than in our cohort (median age 6.6 months, IQR 4.0–18.9 months), and more severely ill. Directly after surgery, patients had multiple organ failure [median Pediatric Logistic Organ Dysfunction II (PELODII) score of 11 (IQR 11–20.75)]. Out of 15 predefined metabolites sampled before surgery, eight metabolites (3-d-hydroxybutyrate, acetone, acetoacetate, citrate, lactate, creatine, creatinine, and alanine) were associated with postoperative PELODII and RACHS scores. Of these metabolites, only creatine (no. 168) and creatinine (no.305) differentiated our patients from controls.

Additionally, out of 521 predefined metabolites, 44 metabolites distinguished 26 patients with single ventricle disease aged 2–19 years of age from controls (37). These included acylcarnitines, amino acids and bile acids. Three of these metabolites were also significantly different between patients and healthy controls in our cohort: dodecanedioylcarnitine (no. 93) and two bile acids; taurocholic acid (no. 155) and glycocholic acid (no. 146). Interestingly, while glycocholic and taurocholic acid levels were higher and dodecanedioylcarnitine levels lower in patients compared to controls, we observed the opposite.

Last, in infants undergoing cardiopulmonary bypass surgery, pre and postoperatively, 165 serum metabolites were measured, aiming to discriminate between survivors and non-survivors, as well as to predict length of stay at the intensive care unit (38). These patients had a median Aristotle score of 10.0 (3.0–19.5), indicating medium procedure-adjusted complexity (score ranges between 1.5 and 25). Multiple pathways demonstrated changes between groups, yet, none of their top 15 metabolites matched with our results. Concluding, when looking at these three paediatric studies, there is only little overlap between identified important features. This could be due to differences in age, diagnosis or disease severity as these factors are well known influencers of the metabolome (39, 40).

Although metabolomic studies in children on ACEi are not available, the effect of ACEi on the metabolome was investigated in adults. In a population-based metabolomics study, 517 metabolites in 1,361 individuals were investigated and findings were replicated in another cohort based on 1,964 individuals (11). Patients on ACEi were selected regardless of ACEi indication. There were differences in the concentrations of five dipeptides and three ratios of di- and oligopeptides between ACEi users and non-users. Two of these dipeptides (aspartylphenylalanine & phenylalanylserine) even showed significant associations with BP response and thus qualified as read outs of ACE-activity. However, these metabolites were not considered important features in our cohort, neither for distinguishing responders from non-responders nor for discriminating between patients and controls. Also 2-oxoglutarate, which predicted lisinopril response in adult hypertensive patients (10), was not included in our lists of important features. Possibly, this could be explained by the demographic differences and different ACEi indications (hypertension vs. HF).

While our study provides new insights in the metabolic perturbations in children with CHD using enalapril, there are some limitations. First, the sample size is relatively small with 63 patients and 46 controls. Due to the complexity of metabolomic studies, there are currently no standard methods for sample size determination. Nevertheless, when compared to existing cohorts investigating the metabolome in the paediatric population, we believe, our study included sufficient patients. Besides, it has the advantage of a narrow age range of the included patients. Also, conducting a larger study including a similar population is challenging as the prevalence of CHD patients using ACEi is relatively low and high quality sampling and analysis for RAAS markers is difficult in a clinical setting (12). As blood for metabolomic analysis in controls was withdrawn after these children were sedated for a minor surgical procedure, this could be considered another limitation. Even though controls were healthy and age-matched, there could be a distorting effect of the sedation they received. Most frequently, anaesthesia was induced with sevoflurane or propofol/fentanyl, of which the effect on the systemic metabolome is still uncertain. In 500 adult surgical patients with high end-tidal sevoflurane concentrations, levels of L-glutamine, pyroglutamic acid, sphinganine and L-selenocysteine were elevated compared to patients with low concentrations (41). In our population, however, concentrations of these metabolites in controls were not elevated, making a sedation-induced effect on the metabolome unlikely. Last, as we used an untargeted metabolomic approach, we could not make the distinction between isobaric compounds. For instance, aldosterone could be replaced by metabolites having a similar m/z ratio that include cortisone or 19-Oic-deoxycorticosterone. Although we cannot identify the exact metabolite that has a distinctive value, we assessed the possible association of all isobaric compounds with cardiovascular disease. This did not lead to different findings and conclusions.

Concluding, the extensive, untargeted metabolomic analysis described here in children with CHD is novel and will allow further studies aiming at disentangling variability in treatment response. Although our initial aim was to distinguish responders from non-responders, we believe, our results indicate that, based on metabolite levels, children with more severe HF exhibit a more profound decrease in their aldosterone levels to enalapril therapy than children with less severe HF. Also, there were very clear differences in metabolic profile between patients and controls, which may help to better understand the pathobiology of paediatric CHD related HF and to develop predictive biomarkers. Ultimately, these metabolites could be valuable in terms of monitoring disease progression.

## Data Availability

The datasets presented in this study can be found in online repositories. The names of the repository/repositories and accession number(s) can be found in the article/Supplementary Material.

## Supplementary material

Supplementary Table S1 Important features for distinguishing responders from non-responders.

Supplemetary Table S2 Important features for distinguishing patients from controls.
